# Heart rate variability in non-apneic snorers and controls before and after continuous positive airway pressure

**DOI:** 10.1186/1471-2466-5-9

**Published:** 2005-07-27

**Authors:** Gregory J Gates, Susan E Mateika, Jason H Mateika

**Affiliations:** 1 Departments of Internal Medicine and Physiology, Wayne State University School of Medicine, Detroit, MI, USA; 2 Research and Development, John D. Dingell Veterans Administration Medical Center, Detroit, MI, USA; 3 Department of Biobehavioral Sciences, Teachers College, Columbia University, New York, NY, USA

## Abstract

**Background:**

We hypothesized that sympathetic nervous system activity (SNSA) is increased and parasympathetic nervous system activity (PNSA) is decreased during non-rapid eye movement (NREM) sleep in non-apneic, otherwise healthy, snoring individuals compared to control. Moreover, we hypothesized that these alterations in snoring individuals would be more evident during non-snoring than snoring when compared to control.

**Methods:**

To test these hypotheses, heart rate variability was used to measure PNSA and SNSA in 11 normotensive non-apneic snorers and 12 control subjects before and 7-days after adapting to nasal continuous positive airway pressure (nCPAP).

**Results:**

Our results showed that SNSA was increased and PNSA was decreased in non-apneic snorers during NREM compared to control. However, these changes were only evident during the study in which snoring was eliminated with nCPAP. Conversely, during periods of snoring SNSA and PNSA were similar to measures obtained from the control group. Additionally, within the control group, SNSA and PNSA did not vary before and after nCPAP application.

**Conclusion:**

Our findings suggest that long-lasting alterations in autonomic function may exist in snoring subjects that are otherwise healthy. Moreover, we speculate that because of competing inputs (i.e. inhibitory versus excitatory inputs) to the autonomic nervous system during snoring, the full impact of snoring on autonomic function is most evident during non-snoring periods.

## Background

Epidemiological findings have suggested that snoring is an independent risk factor for the development of daytime hypertension [1-3]. Moreover, studies completed in normotensive individuals suffering from obstructive sleep apnea have shown that increases in sympathetic nervous system activity (SNSA) precede the development of hypertension [4]. Given these findings, we hypothesized that nocturnal increases in SNSA and decreases in parasympathetic nervous system activity (PNSA) may exist in non-apneic normotensive snoring individuals. Additionally, we postulated that the impact of snoring on autonomic nervous system activity might be most evident during periods of non-snoring (i.e. either during wakefulness or during periods of non-snoring during sleep). This latter postulation was based on findings from humans during wakefulness [5-7] or sleep [8,9], which showed that breathing frequency [5-7,9], pattern (i.e. inspiratory and expiratory time) [5-7,9] and tidal volume [5,7,8] are altered in response to either snoring [9], the application of high frequency oscillations (i.e. simulated snoring) [7,8] or breathing against an increased airway resistance [5,6]. Moreover, these alterations in breathing are accompanied by enhanced PNSA [6] or decreased SNSA [10]. Thus, we postulated that snoring elicits a two-fold response from the autonomic nervous system. An acute response that occurs concomitantly with snoring, in which increases in SNSA and decreases in PNSA may not be clearly evident, and a longer lasting potentially permanent response in which increases in SNSA and decreases in PNSA are unmasked in the absence of snoring. To test these hypotheses, we used the technique of heart rate variability to measure PNSA and SNSA in non-apneic snorers and controls during non-rapid eye movement (NREM) sleep before and after the application of nasal continuous positive airway pressure (nCPAP).

## Methods

Eleven self-reported snoring subjects with no known medical conditions and 12 non-snoring control subjects were recruited from the community. The snoring group was comprised of 9 males (1 Asian, 1 African-American, 2 Hispanic and 5 Caucasian) and 2 Caucasian females (Table 1). The control group was comprised of 10 males (1 Asian, 1 African-American, 2 Hispanic and 6 Caucasian) and 2 Caucasian females (Table 1). All subjects gave their informed consent to participate in the study, which was approved by the Institutional Review Boards of Teachers College, Columbia University, Wayne State University and John D. Dingell VA Medical Center.

**Table 1:** Anthropometric and blood pressure measures

Control						Snorer					
Subject	Sex	Age	BMI	MAP Awake	MAP NREM	Subject	Sex	Age	BMI	MAP Awake	MAP NREM
1	M	28	26.9	95	77	1	M	25	28.1	84	84
2	M	31	26.7	89	88	2	M	35	23	88	88
3	M	25	27.3	83	81	3	M	27	28	70	65
4	F	43	22.5	75	69	4	F	43	25.6	72	62
5	M	33	28	87	83	5	M	36	30	85	82
6	M	27	31	75	74	6	M	34	30	78	75
7	M	27	28.4	85	74	7	M	36	28	88	80
8	M	29	24.5	89	86	8	M	26	23	95	75
9	F	25	20	85	71	9	F	27	24	79	71
10	M	32	27	80	83	10	M	38	26	91	89
11	M	28	23.2	87	80	11	M	28	24.1	88	82
12	M	26	22.9	81	72						
											
**Mean**		**29.5**	**25.7**	**84.3**	**78.2**	**Mean**		**32.3**	**26.3**	**83.5**	**77.5**
**S.E.**		**1.4**	**0.9**	**1.7**	**6.2**	**S.E.**		**1.8**	**0.8**	**2.4**	**2.7**

The snoring and control subjects visited the sleep laboratory on three occasions. Twenty-four hours prior to each occasion the subjects were advised to avoid alcohol and caffeine. During the first visit to the laboratory, which is referred to from hereon as the preliminary study, subjects received a physical examination, which included three separate measures of blood pressure using a standard mercury sphygmomanometer that were separated by 15 minutes. Additionally, subjects completed a general health questionnaire to confirm the absence of pre-existing medical conditions. Subsequently, subjects completed a sleep study in order to familiarize themselves with the laboratory environment and to confirm that the subjects were non-apneic snoring or non-snoring individuals. Moreover, to confirm that subjects were normotensive, beat-to-beat measures of blood pressure were obtained for 1 hour during wakefulness and throughout NREM sleep (see *Nocturnal polysomnography for further details*). The blood pressure measures were in addition to the values obtained during wakefulness using the sphygmomanometer. If subjects suffered from another sleep disorder (i.e. obstructive sleep apnea, insomnia or upper airway resistance syndrome) they were excluded from the study. Individuals with upper airway resistance syndrome were excluded because we were interested in investigating the impact of snoring on heart rate variability independent of excessive cortical arousal from sleep.

The second study was completed in order to measure the autonomic variables outlined below during NREM sleep. This second study will be referred to as Trial 1 from hereon. Subsequent to the completion of Trial 1, the snoring and control subjects adapted to 5 cmH_2_O of nasal continuous positive airway pressure (nCPAP) for 7 days at home. The selection of the nCPAP pressure was based on results from our pilot data, which showed that this level of pressure effectively eliminated snoring in non-apneic individuals. The purpose of the adaptation period was to ensure that the subjects were able to tolerate nCPAP for a minimum of 4 hours during completion of a third sleep study (Trial 2). Nasal continuous positive airway pressure was employed initially for 1 hour during the first night at home. Thereafter, the duration of treatment was increased by an additional hour each night until 4 hours of nCPAP was tolerated. During the adaptation period subjects received a phone call on days 3 and 6 to ensure that the protocol was being followed. During all sleep studies subjects were required to sleep in the supine position. The subjects were monitored via an infrared camera to ensure that this position was maintained throughout the sleep period.

### Nocturnal polysomnography

The sleep monitoring montage included an electroencephalogram (C3/A2, C4/A1, O1/A2, O2/A1), electrooculogram, submental and tibialis anterior electromyogram and an electrocardiogram. Abdominal movements were monitored using a piezoelectric band (Pro-tech, Woodinville, WA) and nasal pressure was measured using a pressure transducer/airflow sensor (Pro-tech, Woodinville, WA). Thus, breathing frequency was monitored breath-by-breath. Oxygen saturation was measured using a pulse oximeter (Biox 3700, Ohmeda Corp., Boulder, CO). Snoring was measured using a microphone that was mounted on the wall located adjacent to the subjects head. During the preliminary study, blood pressure was monitored continuously and non-invasively from the third finger of the left hand using a digital infrared photoplethysmograph (Finapres 2300, Ohmeda Corp., Madison, WI). The accuracy of the blood pressure monitor was verified during pre-sleep wakefulness and nocturnal awakenings by comparing its values to measurements made with a standard mercury sphygmomanometer. To ensure that the monitoring site of the Finapres was adequately perfused with blood throughout the evening, the operation of the Finapres was discontinued consistently during rapid eye movement sleep (REM) and at times during NREM sleep if necessary. The total number of segments analyzed for each subject represented on average 2.2 hours of data obtained from stage II and SWS recorded over the entire sleep period.

During sleep all physiological variables were analogue to digitally converted at a sampling frequency of 200 Hz/channel and input into a microcomputer using a commercially available software package (Gamma, Version 4.0, Astro-Med Inc., West Warwick, RI).

### Data analysis (sleep variables)

Sleep was staged in 30-s epochs according to standard criteria [11,12]. For each subject the total sleep period time as well as the percent of total sleep time spent in a given sleep stage was calculated. The total number of arousals, apneas, hypopneas, snores, and the mean, minimal and maximal oxygen saturation measured was calculated for the total sleep time. An apnea was defined as the absence of inspiratory airflow for a minimum of 10 s. The apnea index was defined as the total number of apneas per hour of sleep. A hypopnea was defined as greater than a 50 % reduction in the flow signal lasting more than 10 s, accompanied by a 2 % decrease in oxygen saturation. We chose to employ a 2 % oxygen desaturation criteria (rather than 3 or 4%) in order to ensure that snoring associated with small changes in oxygen saturation were identified. Based on the use of this criterion we reasoned that if the number of breathing events was similar between snoring and control subjects and less than 5, we could be reasonably confident that the subjects recruited for this study were non-apneic. The hypopnea index was defined as the total number of hypopneas per hour of sleep time. A breath characterized by respiratory noises that registered as an obvious deflection from the baseline of the snoring channel was counted as a snore. In addition, the respiratory noises were subjectively determined to be snores by a polysomnographic technologist monitoring an audio-visual system. We are confident that the sounds recorded were associated with snoring since normal and heavy breathing during wakefulness did not register on the sound system while simulated snoring during wakefulness was detected.

After staging the sleep studies completed during trial 1 and 2, we randomly selected a 15-minute snoring segment from NREM sleep (stage 2 or SWS) recorded between midnight-2 am, 2–4 am and 4–6 am. Thus, 3–15 segments were selected from sleep studies completed during Trials 1 and 2. The segments selected were devoid of apneas, hypopneas and arousals. The snoring segments (Trial 1) were identified as such if 67–100 % of the breaths in a given segment were associated with snoring. Three segments were selected from each study to ensure that our findings reflected the impact of snoring on heart rate variability throughout the night. However, we did not report the results obtained from the snoring segments selected from the beginning, middle and end of the night separately. Although the data was analyzed originally in this fashion, we found that our findings were not time dependent, and thus the data from the three snoring segments were combined.

In addition to the snoring segments, we also analyzed one 15-minute non- snoring segment recorded during Trial 1. In most subjects, only one 15-minute non-snoring segment in deep stage II or SWS was available for analysis. We chose to analyze a non-snoring segment to compare heart rate variability measures in snoring subjects during non-snoring, which was independent of nCPAP application, and snoring.

### Data analysis (respiratory, cardiovascular and autonomic variables)

The number of snores and breaths was calculated for each segment. Subsequently, the values calculated were divided by the total segment time and reported as snoring frequency (S_f_) and breathing frequency (B_f_) (snores/min and breaths/min, respectively). To obtain a measure of SNSA and PNSA the R waves of the electrocardiogram were identified using a threshold detection program. The time intervals between the detected R waves (interbeat interval – IBI) were plotted and the mean IBI was calculated for each segment. Prior to frequency analysis, each 15-minute segment was divided into three 5-minute segments and the interbeat intervals were re-sampled at 20 Hz, which provided 6000 equidistant data points per 5 minute segment [13]. For each 5-min segment, an auto power spectrum was created via a Fast Fourier transform of the interpolated IBI data. The resulting spectrum was integrated and areas associated with discrete frequency bands were determined. Power spectra within the 0.04–0.15 Hz range are considered low frequency components (LF), whereas frequencies of 0.15–0.4 Hz are defined as high frequency components (HF). It is assumed that the power content of the HF component represents PNSA whereas the power content in the LF domain (0.04 – 0.15 Hz) represents SNSA and PNSA at the sinus node [13,14]. The ratio of LF/HF is assumed to be a measure of SNSA.

The absolute values of LF and HF were ln transformed so that the data was normally distributed. Additionally, to minimize the effect that changes in total power have on the absolute values of the low and high frequency power, the LF and HF values were normalized [13]. The presentation of LF and HF values in normalized units represents the relative value of each power component in proportion to the total power minus the very low frequency component. Once the very low frequency component is subtracted, the remaining power equals LF+HF. Thus,

normalized LF = LF ÷ (LF + HF)

normalized HF = HF ÷ (LF + HF)

The normalized units assume a balanced behavior of the sympathetic (normalized LF) and parasympathetic nervous system (normalized HF values). Statistical analysis was completed on both the ln transformed and normalized values (*see Statistical analysis below*). However, for the sake of clarity and based on the guidelines that LF and HF values should be corrected for total power, the normalized data was used as our point of reference in the discussion (see critique of the methods for further discussion of this point).

### Statistical analysis

Unpaired t-tests were used to compare the anthropometric data between groups. A two-way analysis of variance in conjunction with Student-Newman-Keuls post hoc test was used to determine if differences in mean arterial blood pressure existed between groups during wakefulness and NREM sleep during completion of the preliminary study. The two factors in the design were "subject population" (i.e. snorers versus controls) and "arousal level" (i.e. wakefulness vs. sleep). A similar analysis was employed to determine if differences in ln LF, ln HF, LF/HF, normalized LF and normalized HF existed between groups. The two factors in the design were "subject population" (i.e. snorers versus controls) and "treatment" (i.e. before versus after nCPAP treatment). This analysis was also used to determine if differences in sleep architecture existed between and within groups before and after nCPAP treatment. A paired t-test was used to determine whether ln LF, ln HF, LF/HF, normalized LF and normalized HF during non-snoring in trial 1 was different from measures obtained during snoring in trial 1. All values in the tables are presented as means ± SE and the level of significance chosen was p ≤ 0.05.

## Results

Age, body mass index and blood pressure measured during wakefulness and sleep throughout the preliminary sleep study, were not significantly different between the snoring and control groups (Table 1). Additionally, apnea index, hypopnea index, arousal index and mean nocturnal oxygen saturation were not different between the snoring and control groups during Trial 1 (Table 2). Similarly, sleep efficiency and the percentage of time spent in a given stage of NREM sleep did not vary significantly between groups during completion of Trial 1 or Trial 2 (Table 2). Moreover, the sleep architecture during Trial 2 did not differ significantly from that reported during Trial 1 within both groups (Table 2).

**Table 2:** Sleep Measures

	Control(n = 12)		Snorer (n = 11)	
	NREM sleep (without CPAP) (Trial 1)	NREM sleep (with CPAP) (Trial 2)	NREM sleep (without CPAP) (Trial 1)	NREM sleep (with CPAP) (Trial 2)
Apnea index (apnea/hr)	0.38 ± 0.15	0.67 ± 0.34	1.19 ± 0.56	0.16 ± 0.07
Hypopnea index (hypopnea/hr)	1.80 ± 0.71	0.89 ± 0.29	2.14 ± 0.62	0.34 ± 0.19
Arousal index (arousals/hr)	12.87 ± 1.39	12.87 ± 1.02	14.11 ± 2.20	10.74 ± 1.83
Nocturnal Oxygen Saturation (%)	96.58 ± 0.23	96.7 ± 0.30	96.10 ± 0.39	96.40 ± 0.42
Sleep efficiency (%)	88.76 ± 3.62	88.09 ± 2.02	86.32 ± 2.18	83.73 ± 1.99
% of time in Stage I	11.29 ± 1.83	7.83 ± 0.90	7.22 ± 1.04	8.88 ± 1.57
% of time in Stage II	46.04 ± 1.42	45.53 ± 1.91	48.07 ± 2.58	48.48 ± 2.57
% of time in Slow Wave Sleep	22.45 ± 3.10	20.65 ± 1.42	21.95 ± 1.41	21.27 ± 2.36

Snoring episodes measured from the non-apneic snorers were characterized by an average snoring frequency of 11.60 ± 0.43 snores per minute. In contrast, no snoring was detected from the control group. The application of nCPAP effectively eliminated snoring in all subjects.

Breathing frequency and heart rate were not significantly different between or within groups for all conditions during Trial 1 and 2 (Table 3). The two-way analysis of variance revealed that a significant "subject population" (i.e. snorers versus controls) × "treatment" (i.e. before versus after nCPAP treatment) interaction existed for both ln HF and normalized HF. Post-hoc analysis revealed that ln HF and normalized HF recorded during snoring periods were not significantly different from control during trial 1(i.e. before nCPAP treatment) (ln HF - p = 0.47; normalized HF - p = 0.34, respectively) (Table 3). In contrast, normalized HF recorded during nCPAP treatment (i.e. trial 2) in the snoring population was lower than normalized HF recorded from the control population during nCPAP treatment (p = 0.01) (Table 3). Moreover, ln HF and normalized HF recorded during nCPAP treatment (i.e. trial 2) in the snoring population was significantly less compared to similar measures obtained from snoring periods during trial 1 (ln HF - p < 0.0001; normalized HF - p = 0.001) (Table 3).

**Table 3:** Autonomic measures obtained from controls and non-apneic snorers during wakefulness and non-rapid eye movement sleep

	Control (n = 12)		Snorer (n = 11)		
	NREM sleep (without CPAP) (Trial 1)	NREM sleep (with CPAP) (Trial 2)	NREM sleep (without CPAP) (snoring) (Trial 1)	NREM sleep (without CPAP) (non-snoring) (Trial 1)	NREM sleep (with CPAP) (Trial 2)

Heart rate (beats/min)	55.25 ± 1.53	54.58 ± 1.90	57.27 ± 1.45	55.18 ± 1.77	56.27 ± 1.74
Breathing Frequency (breaths/min)	14.89 ± 0.53	14.35 ± 0.46	13.85 ± 0.69	13.91 ± 0.53	14.23 ± 0.62
ln LF	7.24 ± 0.16	7.11 ± 0.13	7.68 ± 0.16	7.99 ± 0.27	7.41 ± 0.19
ln HF	7.99 ± 0.21	7.72 ± 0.19	8.19 ± 0.13	7.83 ± 0.18^§^	7.40 ± 0.21^§^
LF/HF	0.57 ± 0.12	0.70 ± 0.14	0.69 ± 0.08	1.43 ± 0.29^§^	1.20 ± 0.18*
HF (nu)	67.51 ± 3.60	64.44 ± 3.95	62.46 ± 2.60	46.71 ± 5.38^§^	50.32 ± 4.18*
LF (nu)	34.27 ± 4.37	39.24 ± 5.01	36.61 ± 2.58	52.0 ± 5.26^§^	48.79 ± 4.09*

* – significantly different from snorer – without CPAP – snoring; control – with CPAP

§ – significantly different from snorer – without CPAP – snoring

A significant "subject population" (i.e. snorers versus controls) × "treatment" (i.e. before versus after nCPAP treatment) interaction also existed for normalized LF and LF/HF. Post-hoc analysis showed that normalized LF and LF/HF recorded during snoring was not significantly different from control during trial 1 (normalized LF - p = 0.36; LF/HF - p = 0.54, respectively) (Table 3). In contrast, normalized LF and LF/HF recorded during nCPAP treatment (i.e. trial 2) in the snoring population was greater than measures recorded from the control population during treatment (normalized LF - p = 0.01; LF/HF - p = 0.01), and from similar measures recorded from snoring periods during trial 1 (normalized LF - p < 0.001; LF/HF - p < 0.001) (Table 3).

Within the snoring group during trial 1, ln HF (p = 0.03) and normalized HF (p = 0.01) were less and LF/HF (p = 0.02) and normalized LF (p = 0.01) were greater during non-snoring as compared to snoring (Table 3).

## Discussion

### Summary of findings

Based on the normalized HF and LF values our major findings are that PNSA and SNSA during snoring (i.e. Trial 1) was not significantly different from measures acquired from non-snoring control subjects. However, PNSA was decreased and SNSA was increased in the snoring population compared to control when snoring was abolished with nCPAP (i.e. Trial 2). Similarly, PNSA was less and SNSA was greater when snoring was abolished (i.e. non-snoring periods during trial 1 or during nCPAP application in trial 2) compared to when it was present (trial 1) within the snoring population. Finally, within the control group PNSA and SNSA did not vary before and after treatment with nCPAP.

### Critique of the methods

Our subjects were exposed to nCPAP for a 7-day adaptation period prior to completing Trial 2. This adaptation period occurred after a baseline study was completed. Consequently, the baseline and treatment study were not randomized. Nevertheless, if the order of the trials impacted on our measures we would expect that the response to nCPAP application would have been similar between groups since both groups were comparable (e.g. healthy, no complaint of daytime sleepiness) in all aspects with the exception of snoring. This was not the case.

It is also unlikely that any differences observed between groups during Trial 2 were a consequence of discomfort associated with being unfamiliar with nCPAP treatment. Both the snoring and control subjects adapted to the treatment for a similar duration of time prior to participating in Trial 2, and no significant differences in heart rate variability occurred within the control group when comparing measures from Trial 1 and 2. Moreover, it is also improbable that the differences observed were a consequence of discrepancies in nCPAP compliance because, based on subject self-report, this measure was similar between groups. Although it is possible that some subjects did not truthfully report their compliance, we believe it is unlikely that healthy non-snorers and non-apneic snorers would have tolerated nCPAP for at least 4 hours during Trial 2 if they did not adhere to the protocol during the adaptation period. The ability of the subject's to tolerate nCPAP is supported by the measures of sleep architecture, which were similar between trials 1 and 2. Lastly, it is doubtful that the impact of nCPAP on cardiovascular function [15], and consequently autonomic function, was different between groups since the applied pressure was identical (5 cmH_2_O in all cases) for both groups.

Heart rate variability has been used in numerous studies to obtain non-invasive measures of SNSA (LF/HF and normalized LF) and PNSA (HF and normalized HF). The changes in the HF domain that we observed in our snoring subjects were likely indicative of changes in PNSA since this assumption has been well established by a number of physiological investigations [13]. In contrast, questions remain regarding the validity of LF/HF and normalized LF as a non-invasive indicator of SNSA [14]. One issue is that the LF/HF ratio assumes that HF in the denominator (i.e. PNSA) cancels out the influence of PNSA in the low frequency domain of the power spectrum, leaving the LF/HF ratio as a measure of SNSA. Although we assume that LF/HF and normalized LF are indicative of changes in SNSA we acknowledge that the support for this assumption is equivocal. Nonetheless, we have included this analysis along with the HF measures for those who accept that LF/HF or normalized LF reflects SNSA.

Investigators have shown that absolute values of LF and HF are impacted by changes in total power [13]. Consequently, it has been suggested that absolute LF and HF values should be normalized based on changes in total power [13]. Based on this guideline, and for the sake of clarity, we have used normalized LF and HF as our reference point in the subsequent discussion of the results. Nevertheless, independent of whether one accepts that normalization of our data is necessary, results obtained from the normalized LF (i.e. SNSA) and HF (i.e. PNSA) data are similar to the ratio of absolute LF/HF (i.e. SNSA) and ln HF (i.e. PNSA).

### Parasympathetic and sympathetic nervous system activity during snoring and non-snoring

Parasympathetic nervous system activity and SNSA during snoring (i.e. trial 1) were not significantly different from measures obtained from control subjects. This finding on its own implies that snoring does not influence autonomic function. However, our results showed that PNSA was less and SNSA was greater in the snoring group compared to control when snoring was abolished during trial 2. Similarly, PNSA was less and SNSA was greater in the snoring group when snoring was abolished compared to when snoring was present.

These latter two findings have three primary implications. The first implication is that snoring may induce an acute increase in PNSA and a decrease in SNSA, which may mask the more deleterious effect that snoring has on autonomic function. This would account for the similarities in autonomic function observed between groups during trial 1 despite the differences that were present during trial 2. The second implication is that the deleterious long-lasting impact that snoring may have on autonomic function (*see Physiological Mechanisms for additional discussion on this issue*) is more evident during periods of non-snoring as indicated by the differences observed between the groups during trial 2. The third implication is that short-term nCPAP treatment did not eliminate this long-lasting impact. Arguably, SNSA might have decreased and PNSA increased because of nCPAP treatment. However, the lack of response to treatment is not surprising since the subjects did not receive treatment for a prolonged period with respect to number of days and nighttime hours of exposure.

### Physiological mechanisms

We can only speculate on the mechanism responsible for the potential decrease in SNSA and increase in PNSA that occurs during snoring. Snoring is often associated with increased airway resistance and the development of more negative intrathoracic pressures [9]. Additionally, snoring, or the application of high frequency, low amplitude pressure oscillations (which simulates snoring), has been associated with i) decreases in breathing frequency [5,6,9] ii) increases in inspiratory time or inspiratory time/total time ratio [5,6,9] and iii) increases in tidal volume [5,7,8,16]. Moreover, breathing frequency, breathing pattern or tidal volume are known to impact on measures of heart rate variability [13,14]. Thus, it is possible that the increase in HF that we observed during snoring, which mirrors the amplitude of respiratory sinus arrhythmia, was caused by changes in one or more of these variables. Since snoring did not alter breathing frequency in our study, we speculate that alterations in pattern and/or tidal volume may have contributed to the increase in measures of HF. More specifically, prolongation of inspiratory time coupled with an increase in tidal volume may have led to increased activation of pulmonary vagal afferents, which have a profound influence on respiratory sinus arrhythmia, and hence measures of HF. Additionally, activation of pulmonary vagal afferents are known to inhibit SNSA [17], thus the decrease in SNSA during snoring may have been a consequence of this activation.

Since snoring itself was not accompanied by an increase in SNSA or decrease in PNSA compared to control, the question remains as to how snoring may ultimately result in long-lasting increases in SNSA and/or decreases in PNSA during non-snoring periods. There are at least three possibilities. First, it is possible that snoring subjects participating in our study were genetically predisposed to a reduction in PNSA and an increase in SNSA. Given our experimental design, we cannot eliminate this possibility. Future studies designed to examine autonomic nervous system activity in non-apneic snorers, before and after prolonged nCPAP treatment, may resolve whether the changes in PNSA and SNSA we observed are an inheritable trait.

Second, the impact of exposure to nCPAP treatment on the non-apneic snorers compared to controls may have been different (see *Critique of methods *for detailed discussion on this issue). We consider this possibility highly unlikely since the nCPAP pressure used and the duration of application was similar for both populations. More importantly, even in the absence of nCPAP treatment, PNSA decreased and SNSA increased during non-snoring compared to snoring (i.e. non-snoring vs. snoring in trial 1).

Third, we postulate that inputs activated during snoring concurrently with vagal afferents may ultimately be responsible for the long-lasting changes in PNSA and SNSA activity that we observed. Thus, the influence of pulmonary vagal afferents may predominate during snoring, leading to a reduction in SNSA and an increase in PNSA. However, we speculate that resistive loading and snoring may concurrently enhance brainstem activity (i.e. the reticular activating system), possibly via inputs from mechanoreceptors [18], metabolic receptors [10,19], and upper airway receptors [20,21], that eventually leads to increases in SNSA and decreases in PNSA once snoring is terminated. The possibility that snoring may influence brainstem activation and ultimately autonomic nervous system activity is supported by the findings of Lofaso and colleagues [22]. These investigators showed in non-apneic snorers that graded levels of arousal (based on various changes in EEG characteristics) subsequent to termination of snoring were associated with increases in heart rate and blood pressure. More importantly for our findings, Lofaso and colleagues [22] showed that even in the absence of cortical arousal (no evidence of EEG changes) blood pressure and heart rate were greater immediately after termination of snoring compared to measures obtained during snoring [22]. The possibility that both the acute and long-lasting impact of brainstem activation is masked during snoring, when pulmonary vagal afferents are most influential, is supported by studies which have shown that muscle sympathetic nervous system activity is profoundly inhibited by the activation of pulmonary vagal afferents in the presence of stimuli known to enhance SNSA [23].

### Clinical implications

The upper airway resistance syndrome has been described as a form of sleep disordered breathing that is characterized by repetitive increases in resistance in airflow within the upper airway leading to brief cortical arousals and daytime somnolence [24]. In contrast to this clinical description, our subjects did not display excessive numbers of cortical arousal compared to control and did not suffer from daytime somnolence. Nevertheless, despite the absence of cortical arousal we showed that PNSA was decreased and SNSA activity was increased during non-snoring periods in non-apneic snorers compared to control and during non-snoring periods compared to snoring periods within the non-apneic population. The latter finding is similar to results which showed that blood pressure and heart rate increased at the termination of snoring independent of cortical arousal [22,25]. Thus, alterations in autonomic nervous system activity may lead to autonomic and cardiovascular alterations in individuals with increased airflow resistance independent of evidence of cortical arousal. Moreover, our results suggest that alterations in heart rate variability may precede changes in traditional markers of cardiovascular function (i.e. blood pressure) in non-apneic snoring individuals. Given that measures of heart rate variability have been shown to be useful as a prognostic indicator of future cardiovascular events [13,14,26-30] these measures may be useful in determining whether normotensive individuals that snore (or for that matter suffer from obstructive sleep apnea) are at an increased risk for the development of hypertension or cardiovascular disease. Whether the alterations that we observed in our relatively young, healthy and predominantly male snoring population would benefit from long-term treatment with nCPAP, or become more severe with age and/or the presence of other co-morbid conditions remains to be determined.

## Competing interests

The author(s) declare that they have no competing interests.

## Authors' contributions

GG participated in data acquisition, performed statistical analysis on the data and helped draft the manuscript. SM participated in data acquisition, coordination of the study and helped to draft the manuscript. JM conceived the study, participated in its design and coordination, performed statistical analysis and helped draft the manuscript. All authors read and approved the final manuscript.

## Pre-publication history

The pre-publication history for this paper can be accessed here:


